# Editorial: Genome and metagenome interactions in the plant holobiont for plant health and productivity

**DOI:** 10.3389/fpls.2024.1463795

**Published:** 2024-07-25

**Authors:** Alessandro Passera, Carmen Escudero-Martinez

**Affiliations:** ^1^ Department of Agricultural and Environmental Sciences, University of Milan, Milan, Italy; ^2^ School of Life Sciences, University of Dundee, Dundee, United Kingdom

**Keywords:** plant-associated microbiome, multi-omic analyses, plant-microbe interaction, rhizosphere microbiome, sustainable agriculture

Plants engage with a diverse array of microorganisms, collectively referred to as the plant microbiota. These interactions significantly impact plant health and survival: while some may lead to plant diseases, others can promote growth and resilience (Yu et al., 2019). In agriculture, understanding the mechanisms governing plant-microbe interactions is key to their translation into sustainable agronomic strategies such as biocontrol, biostimulation, biofertilization (Arif et al., 2020), and more recently also in plant breeding for the microbiota interactions (Escudero-Martinez and Bulgarelli, 2019, 2023). Whereas in natural ecosystems, plant-microbe interactions are fundamental to ecosystem functioning by facilitating nutrient cycling, soil health, plant survival, and overall biodiversity (Parasuraman et al., 2019). Hence, plant-microbiota research is key to developing strategies for sustainable agricultural production, food security, and environmental conservation.

Our research aims to contribute to the knowledge in this field (Passera et al., 2020, 2021; Escudero-Martinez et al., 2022; Escudero Martinez, 2023), thus we were determined to open the present Research Topic in Frontiers in Plant Science intending to compile a collection reflecting on the field status. This Research Topic has advanced and consolidated knowledge on how plants actively recruit specific microorganisms from the soil microbiota, influenced by factors such as soil characteristics, plant species, developmental stage, environmental conditions, and agricultural practices. We have attempted likewise to present recent multidisciplinary advances in genomics, metagenomics, metabolomics, and microbiota analyses in 8 insightful manuscripts compiling the research of 69 authors from all around the World.

The plant-associated microbiota is key to the adaptation of wild plants to their natural environments. The study by Liu et al. compared different Camellia species growing either in acidic or karst soils, identifying microorganisms (e.g., *Candidatus* Rokubacteria) specifically enriched in the rhizosphere of Camellia growing in karst soils. These microorganisms might help the plant obtain more nutrients, thus strengthening their secondary metabolism and mitigating the stress associated with adverse karst environments.

The manuscript by Csorba et al. examines how in natural ecosystems the soil type, developmental stage, and geographical location influence the root microbiota and metabolome of the wild medicinal plant *Echium vulgare*. All these variables affected the root metabolome, with sugars and pyrrolizidine alkaloids more abundant at the rosette stage, and echimidine showing location-dependent variations. Microbial diversity varied predominantly by location and soil pH, with acidophilic bacteria being prevalent in acidic soils. Overall, this research illustrates the dynamism and complexities of the multiple interactions of the microbiota with their hosts.

In agricultural environments, agronomic strategies such as tillage, fertilisation, and irrigation have implications for the crop’s microbiota (Romano et al.) This research piece assesses the above-mentioned agronomic practises on winter wheat yield, nitrogen uptake, microbial activity, and rhizosphere-root microbial communities. Wheat yield and nitrogen uptake responded to irrigation, fertilization, and genotype, while microbial activity varied with irrigation and tillage. This research informs sustainable agricultural practices by linking them to microbial community dynamics.

Another important agronomic strategy linked to the microbiota is the application of microbes as biofertilizers (Bhardwaj et al., 2014). Li et al. investigated the mechanism by which a bacterial strain increased cash crop growth. The authors showed an increase in soil fast-acting nitrogen and phosphorus, suggesting an enhanced microbial nutrient turnover. This was confirmed by nitrogen cycling genes favouring ammonium oxidation. Metagenome analysis revealed microbial genomes putatively involved in plant growth promotion traits. These findings underline the biofertilizers’ potential to optimize nutrient availability for plants.

An additional use for plant’s beneficial microbes is the prevention or mitigation of environmental stresses. Senizza et al. investigated changes in the microbiota and metabolic profile of the model plant *Arabidopsis thaliana* treated with *Trichoderma* spp. and challenged with heat and drought stresses. In this study treatment with *Trichoderma* triggers a restructuring of microbial communities and plant’s metabolic profiles. By the integration of multi-omics analyses, the study identified a plant growth response that is mediated by the entire plant holobiont, even under stress conditions.

Another research in *A. thaliana* microbiota investigates the mechanistic relationship between genotype and leaf microbiota. In this study by Li et al., network mapping was used to dissect the leaf-microbiota interactions finding interactions that are consistent with different ecological theories, including the golden threshold and surrender-resistance hypotheses. This study reveals the “endophenotype” of microbial networks linking plant genotype and phenotype.

The plant immune system shapes plant-microbiota interactions (Trivedi et al., 2020). Oldstone-Jackson et al. investigated the impact on the microbiota of transmembrane pattern recognition receptors (PRRs) mutants in *A. thaliana* roots. PRRs are important components of the plant immune system implicated in triggering the plant defence cascade upon recognition of microbe-associated molecular patterns (MAMPs). The authors found a limited impact of these mutants on root endophytic bacterial and fungal microbiota composition under field conditions. Suggesting that, at least under field conditions, the challenge of balancing pathogen recognition with the recruitment of beneficial microbiota in roots requires further investigation.

Lastly, Wang et al. investigated the post-harvest soil microbiota of different bean species (legumes) used for intercropping in tea plantations. While the use of intercropping generally enhanced the quantity of nutrients found in the soil, the different legume species recruited unique microbial communities with the adzuki bean being associated with a lower pH and the presence of weeds.

Overall, the Research Topic highlights the importance of the interactions between plants and their microbiota in both natural and agricultural environments. Factors like soil characteristics, plant species, genetics, developmental stages, and agricultural practices, shape these communities. It is therefore important to take a step forward and resolve the significance of this microbial temporal and spatial dynamics. Would functions rather than taxonomies provide more consistent insights across environments? Which microbes are actively recruited by host plants during stress responses? Upon stress, do microbes confer beneficial functions, or are opportunistic microbes also present, and if so, what dictates the plant response outcome? Can stresses enhance positive plant-microbe symbiosis in the form of beneficial plant feedback? Ultimately, this Research Topic aimed to enlighten the interactions that influence plant health, resilience, and productivity, offering promising avenues for sustainable and equitable agriculture.

## Author contributions

AP: Writing – original draft, Writing – review & editing. CE-M: Writing – original draft, Writing – review & editing.
